# Participant retention in follow-up studies of prematurely born children

**DOI:** 10.1186/s12889-019-7575-6

**Published:** 2019-09-06

**Authors:** Victoria MacBean, Simon B. Drysdale, Sanja Zivanovic, Janet L. Peacock, Anne Greenough

**Affiliations:** 1 Centre for Human Performance, Exercise and Rehabilitation, Brunel University London, London, UK; 2 Oxford Vaccine Group, Department of Paediatrics, University of Oxford, Oxford, UK; 3 Department of Paediatrics, University of Oxford, Oxford, UK; 4 School of Population Health and Environmental Sciences, Faculty of Life Sciences & Medicine, King’s College London, London, UK; 5 NIHR Biomedical Research Centre at Guy’s & St Thomas’ NHS Foundation Trust and King’s College London, London, UK; 6 Department of Women and Children’s Health, School of Life Course Sciences, Faculty of Life Sciences & Medicine, King’s College London, London, UK; 7 The Asthma UK Centre for Allergic Mechanisms of Asthma, King’s College London, London, UK

**Keywords:** Recruitment, Follow-up study, Prematurity

## Abstract

**Background:**

Follow-up studies of infants born prematurely are essential to understand the long-term consequences of preterm birth and the efficacy of interventions delivered in the neonatal period. Retention of participants for follow-up studies, however, is challenging, with attrition rates of up to 70%. Our aim was to examine retention rates in two follow-up studies of prematurely born children and identify participant or study characteristics that were associated with higher attrition, and to discuss retention strategies with regard to the literature.

**Methods:**

Data from children recruited at birth to one of two studies of prematurely born infants were assessed. The two studies were the United Kingdom Oscillation Study (UKOS, a randomised study comparing two modes of neonatal ventilation in infants born less than 29 weeks of gestational age (GA)), and an observational study examining the impact of viral lower respiratory tract infections in infancy in those born less than 36 weeks of GA (virus study). The UKOS participants, but not those in the virus study, had regularly been contacted throughout the follow-up period. UKOS subjects were followed up at 11 to 14 years of age and subjects in the virus study at 5–7 years of age. At follow up in both studies, pulmonary function and respiratory morbidity were assessed. Retention rates to follow-up in the two studies and baseline characteristics of those who were and were not retained were assessed.

**Results:**

Retention was significantly higher in UKOS than the virus study (61% versus 35%, *p* < 0.0001). Subjects lost to UKOS follow up had greater deprivation scores (*p* < 0.001), a greater likelihood of intrauterine tobacco exposure (*p* = 0.001) and were more likely to be of non-white ethnicity (*p* < 0.001). In the virus study, those lost to follow-up had higher birth weights (*p* = 0.036) and were less likely to be oxygen dependent at hospital discharge (*p* = 0.003) or be part of a multiple birth (*p* = 0.048).

**Conclusions:**

Higher retention was demonstrated when there was regular contact in the follow-up period. Both social factors and initial illness severity affected the retention into follow-up studies of prematurely born infants, though these factors were not consistent across the two studies.

## Background

Follow-up studies of infants born prematurely are essential to understand the long-term consequences of preterm birth and the efficacy of interventions delivered in the neonatal period. There are a number of studies whose short-term results have been at variance with important outcomes at follow up [1–3]. Retention of participants for follow-up studies, however, is challenging, with attrition rates of up to 70% [4]. Participant attrition results in loss of statistical power and may introduce bias into study results. Strategies, therefore, are required to mitigate attrition, in order that the value of prospectively recruited cohorts is not lost. We have conducted two studies involving long-term follow-up of cohorts of children born prematurely. Our aims were to compare retention at follow-up of the two studies, highlight methodological differences between the studies’ follow-up design that may have influenced retention, identify any groups at particular risk of attrition, and discuss retention strategies with regard to the literature.

## Methods

Analysis was undertaken of the results of the follow-up study of the United Kingdom Oscillation study (UKOS) and a follow-up study assessing the outcomes of viral infections in infancy of prematurely born infants (virus study). The follow-up studies were approved by the South West London National Research Ethics Service Committee and the National Research Ethics Service Committee West Midlands – Coventry & Warwickshire respectively. Informed, written parental consent was given for all the children to take part in the follow-up studies.

### United Kingdom Oscillation Study (UKOS)

UKOS [5] was a multicentre, randomised trial comparing the efficacy of high frequency oscillatory ventilation to conventional mechanical ventilation initiated within an hour after birth in infants born prior to 29 weeks of gestational age. The UKOS cohort was recruited from 22 centres in the United Kingdom, as well as one centre in each of Ireland, Singapore and Australia. Follow-up, however, was only attempted in children from the UK and Ireland. A subgroup had lung function assessments at 1 year [6] and all were seen by their local paediatrician at 2 years who documented the children’s healthcare utilisation and respiratory symptoms [7]. All UKOS participants were sent birthday and season greeting cards each year. There was also an online presence in the form of a blog (https://ukos.wordpress.com/), which provided parents and children with regular study updates and information about publications from the study.

UKOS participants were asked to attend for follow up at King’s College Hospital NHS Foundation Trust (KCH) at 11 to 14 years when pulmonary function, atopy, environmental tobacco smoke exposure, behavioural outcomes, health-related quality of life and academic achievements were assessed [1].

### Virus study

A single centre, observational study was undertaken at KCH to examine the impact of viral lower respiratory tract infections (LRTIs) in infancy on respiratory morbidity and pulmonary function. Pulmonary function was measured at 1 year of age [8] and healthcare utilisation was recorded over the first year after hospital discharge and health related costs of care calculated [9]. The cohort was then recalled at 5–7 years of age for pulmonary function and healthcare utilisation assessment [10]. Families did not receive any communication from the research team between completion of the initial follow-up study and the invitation to the school-age follow up.

### Analysis

Baseline characteristics of participants in each study who were and were not successfully followed up were compared in order to identify factors that might influence retention. Infant characteristics were selected for comparison as these are known at the enrolment stage and any differences identified would highlight for future authors groups in whom specific retention strategies would be required. The baseline characteristics available for both cohorts (sex, maternal ethnicity, socioeconomic status (index of multiple deprivation (IMD), birth weight, gestational age, whether the infant was part of a multiple birth, whether surfactant had been given, tobacco exposure in utero, whether postnatal steroids had been administered and whether the infant was oxygen dependent at 28 days and at hospital discharge) of the children who were and were not retained to the follow up stage of the studies were compared using unpaired t-tests and Chi square tests (for normally-distributed data) or the Mann-Whitney test and Fisher’s exact test (for non-parametric data). Data analysis was undertaken using SPSS Version 24 (IBM Corp, Chicago, IL).

## Results

**Fig. 1:**
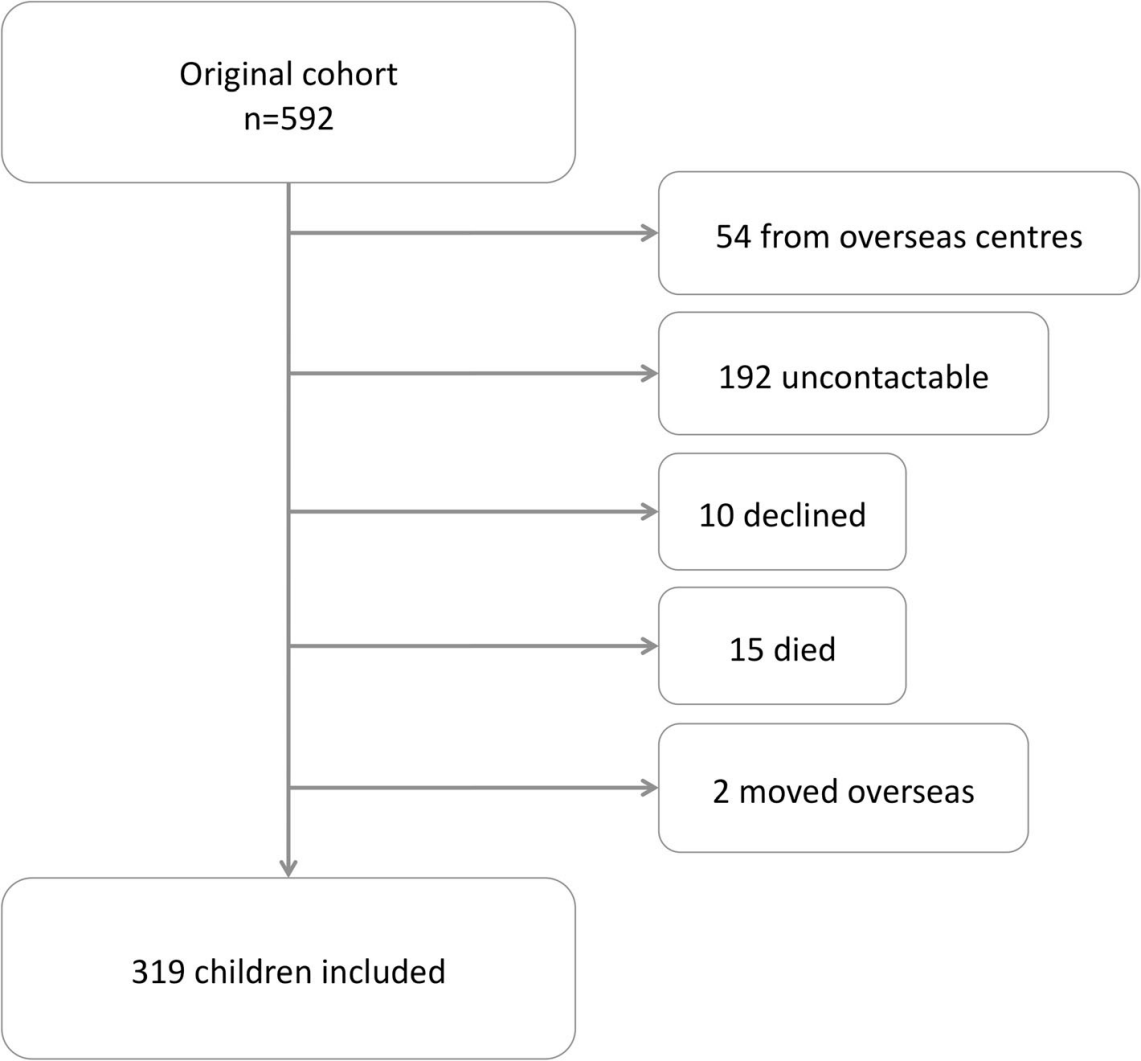
Participant attrition in UKOS for follow-up measurements at 11–14 years of age

**Fig. 2:**
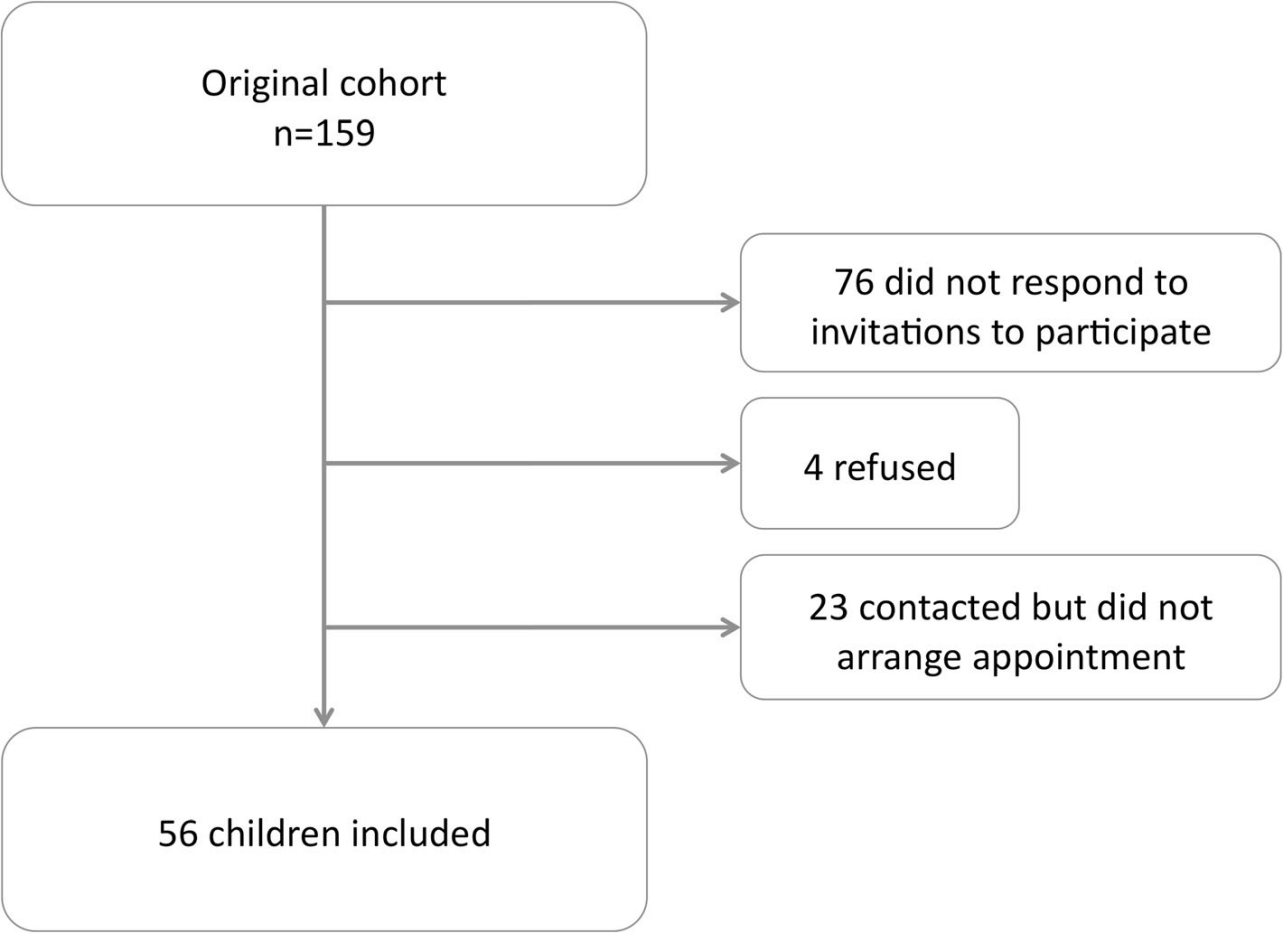
Participant attrition at school age follow up in a study investigating the impact of early life viral lower respiratory tract infections in prematurely born infants

At the UKOS 11 to 14-year follow-up, a total of 592 children who had survived to hospital discharge were eligible for participation, of whom 319 (54%) were studied (Fig. 1). Excluding subjects in whom follow up was not attempted (recruited at an overseas site or died prior to follow-up), the retention rate was 61%. At school-age follow up in the virus study 56 children (35%) were successfully studied (Fig. 2) [10]. The follow-up rate was significantly lower in the virus study compared to UKOS (*p* < 0.0001) as indicated by Chi-squared analysis.

Baseline characteristics of those subjects who were and were not retained in the UKOS follow up study demonstrated significant differences regarding ethnicity, index of multiple deprivation and maternal smoking in pregnancy (Table 1). Non-white ethnicity was more prevalent in those lost to follow-up (*p* < 0.001). Those infants who did not attend follow-up also had significantly higher rates of in utero tobacco exposure (*p* = 0.001) and higher socioeconomic deprivation scores (*p* < 0.001). In the virus study, those not followed up had significantly higher birth weights (*p* = 0.036) and were significantly less likely to be oxygen dependent at discharge (*p* = 0.003) or be a multiple birth (*p* = 0.048, Table 2).

**Table 1:** Characteristics of subjects who were and were not recruited for 11–14 year old follow up in the United Kingdom Oscillation Study

	Followed up (*n* = 319)	Not followed up (*n* = 204)	*p* value
Sex (M: F)	162/319 (51%)	109/204 (53%)	0.55^*^
Maternal ethnicity			
White	285/318 (90%)	149/203 (73%)	Overall
Black	21/318 (6.6%)	35/203 (17%)	< 0.001^*^
Other	12/318 (3.8%)	19/203 (9.3%)	
Index of multiple deprivation (IMD)	15.2 (1.0 to 68.1)	28.2 (1.1 to 70.0)	< 0.001^b^
Birth weight (g)	895 (209)	914 (204)	0.31^a^
Birth weight (z score)	−0.59 (−3.45 to 2.41)	−0.41 (−3.28 to 2.17)	0.050^b^
Gestational age (weeks)	26.9 (1.33)	26.7 (1.39)	0.35^a^
Multiple birth	76/319 (24%)	45/204 (22%)	0.64^*^
Surfactant given	310/319 (97%)	203/204 (99%)	0.097^*^
Maternal smoking in pregnancy	69/292 (24%)	72/188 (38%)	0.001^*^
Postnatal steroids	84/314 (27%)	61/203 (30%)	0.42^*^
Oxygen dependency at 28 days	262/319 (82%)	164/204 (80%)	0.62^*^
Oxygen dependency at hospital discharge	71/315 (23%)	44/204 (22%)	0.80^*^

Data are shown as n (%), median (interquartile range) or mean (SD)

^*^indicates *p* value generated from Chi squared analysis

^a^from unpaired t test

^b^from Mann Whitney u test

**Table 2:** Characteristics of subjects who were and were not recruited for school age follow up in the virus study

	Followed up (*n* = 56)	Not followed up (*n* = 103)	*p* value
Sex (M: F)	28: 28	59: 44	0.378^*^
Maternal ethnicity			
White	23/56 (41%)	32/103 (31%)	Overall
Black	22/56 (39%)	54/103 (52%)	0.278^c^
Other	11/56 (20%)	17/103 (17%)	
Index of multiple deprivation (decile)	3 (2–5)	3 (2–5)	0.838^b^
Birth weight (g)	1836 (707)	1937 (571)	0.036^a^
Gestational age (weeks)	33.86 (30.71–34.86)	33.71 (31.71–35.29)	0.435^b^
Multiple birth	22/56 (39%)	25/103 (24%)	0.048^*^
Surfactant given	13/56 (23%)	20/103 (19%)	0.573^*^
Maternal smoking in pregnancy	8/56 (14%)	17/103 (17%)	0.713^*^
Postnatal steroids	2/56 (4%)	1/103 (1%)	0.250^*^
Oxygen dependency at 28 days	2/56 (4%)	0/103 (0%)	0.054^*^
Oxygen dependency at hospital discharge	11/56 (20%)	5/103 (5%)	0.003^*^

Data are shown as n (%), median (interquartile range) or mean (SD)

^*^indicates *p* value generated from Chi-squared analysis

^a^from unpaired t test

^b^from Mann Whitney u test

^c^from Friedman’s test

Across both studies, there were no differences observed in sex, gestational age, postnatal steroid administration, oxygen requirement at 28 days, or rates of surfactant administration between those infants who were and were not successfully followed up. There were no common characteristics across the two studies in which differences were observed in those who did and did not attend for follow up.

## Discussion

We have shown significantly different success rates in follow up of two studies of prematurely born infants. Comparison of participant characteristics between those who were and were not successfully followed up highlighted that there were no consistent predictive factors across the two studies which identified individuals at higher risk of loss to follow up. In the larger UKOS trial, non-white ethnicity, socioeconomic deprivation and maternal tobacco use were more prevalent in those who did not attend for follow up. In the virus study, lower birth weight, singleton birth and persistent oxygen requirement at hospital discharge were associated with greater follow up participation. In light of the lack of common factors associated with loss to follow up, we suggest that factors related to follow up study design may be more relevant.

We have demonstrated higher retention rates in a cohort with whom there had been regular contact to the follow-up stage [6]. Staff continuity and development of trust has been highlighted as helping to prevent attrition in a longitudinal study of lead-exposed children [11] and in a study of development in children of mothers with a history of substance misuse [12]. In both the studies we report the senior investigators remained the same, but junior researchers who undertook the measurements had changed from those in the original studies. The regular contact with the children and their families with cards and a newsletter in UKOS, we suggest may contribute to the differences in attrition between the two studies.

The factors associated with attrition differed between the two studies. The participants of UKOS were all born less than 29 weeks of gestational age and all had been ventilated from birth. It is then not surprising that the severity of their initial illness did not determine whether they consented to follow-up. Instead the significant risk factors for attrition were social factors, greater deprivation scores and intrauterine tobacco exposure. Previous studies have suggested that indicators of lower socioeconomic status are predictive of attrition in longitudinal studies commencing in infancy [13, 14]. Those data highlight individuals who may require more intense explanation regarding the importance of taking part in the follow-up. In addition, more were of non-white ethnicity which may suggest a need for more tailored support for those whose first language may not be English. In contrast, in the virus study determinants of initial illness severity were significantly related to attrition rate. Infants recruited into the virus study, although born prematurely, were significantly more mature than the UKOS cohort. It is likely those who had a higher birth weight, were not oxygen dependent at discharge or of a multiple birth had a very short time of routine clinical follow-up, which may have led to lower interest in engagement in the later research.

In the paediatric setting, various approaches have been suggested to optimise retention of study participants. Appointments must be flexible around family and work commitments [11, 15] and provision must be made for study participant’s siblings, either by allowing siblings to attend appointments or, if this is not practicable, providing formal childcare [11, 12]. Practical assistance in the form of arranging transport to attend studies (rather than simply reimbursing participants after attendance) and organising onward referrals to address any new incident health needs were further highlighted as being beneficial [11]. Both of our studies welcomed siblings to attend and offered appointments at weekends and during school holidays in addition to weekday appointments. Transport was arranged for all the UKOS participants as the majority lived outside London, whereas for the virus study reimbursement was given for travel, but not arranged, as participants lived within easy reach of the testing location. At the follow-up of both of our studies, the researchers assessed specific health needs and made onward referrals and recommendations as needed, but this was not explicitly advertised in the literature families received prior to recruitment.

The perception of the importance of the research may be a key determinant of choosing to participate [16]. In addition, in community-based clinical trials, it has been identified that participants’ understanding of the study importance was a key determinant of retention [2]. The UKOS blog gave parents updates on publications arising from the study, thereby likely emphasising the success of the research programme.

Obtaining relatives’ contact details as well as those of the participants or parents [17], making interim contact between study visits [18] and providing small financial incentives to update contact information [19, 20] have been suggested as effective methods to reduce attrition due to loss of contact. The annual birthday and seasonal greeting cards sent to the UKOS cohort asked parents to maintain up to date contact details. To undertake such activity carries a cost, thus it is important that additional funding for participant retention that extends beyond the end of the main funding source should be sought [19].

The use of study-specific (rather than institutional) logos may aid study retention [19, 21] by enforcing the identity of the study and may be a stronger reminder of previous participation than written descriptions. The UKOS newsletters regularly featured photographs of the study team, including senior investigators and previous members of the team as well as the study logo.

## Conclusions

In conclusion, retention rates significantly differed between our two studies. Although differences were observed in the baseline characteristics of those lost to follow up versus retained, these factors were not common across the two studies. It seems plausible, therefore, that differences in follow up design explained the differing retention rates. We suggest, based on our experience and findings from the literature, that establishing and reinforcing the study’s identity and purpose to participants, ongoing regular contact with participants beyond the completion of initial study and providing regular feedback on the study may improve retention at follow-up.

## Data Availability

The datasets used and/or analysed during the current study are available from the corresponding author on reasonable request.

## Abbreviations

GA: Gestational age
LRTI: Lower respiratory tract infection
UKOS: United Kingdom Oscillation Study
